# Anti-inflammatory potential of PI3Kδ and JAK inhibitors in asthma patients

**DOI:** 10.1186/s12931-016-0436-2

**Published:** 2016-10-04

**Authors:** Thomas Southworth, Jonathan Plumb, Vandana Gupta, James Pearson, Isabel Ramis, Martin D. Lehner, Montserrat Miralpeix, Dave Singh

**Affiliations:** 1 The University of Manchester; Division of Infection, Immunity & Respiratory Medicine; Manchester Academic Health Science Centre; University Hospital South Manchester NHS Foundation Trust, Southmoor Road, Manchester, M23 9LT UK; 2 Almirall R&D Center, Sant Feliu de Llobregat, Barcelona Spain

**Keywords:** Asthma, Phosphatidylinositol 3-kinase delta, JAK/STAT, T-cell cytokines, Bronchoscopy

## Abstract

**Background:**

Phosphatidylinositol 3-kinase delta (PI3Kδ) and Janus-activated kinases (JAK) are both novel anti-inflammatory targets in asthma that affect lymphocyte activation. We have investigated the anti-inflammatory effects of PI3Kδ and JAK inhibition on cytokine release from asthma bronchoalveolar lavage (BAL) cells and T-cell activation, and measured lung PI3Kδ and JAK signalling pathway expression.

**Method:**

Cells isolated from asthma patients and healthy subjects were treated with PI3Kδ or JAK inhibitors, and/or dexamethasone, before T-cell receptor stimulation. Levels of IFNγ, IL-13 and IL-17 were measured by ELISA and flow cytometry was used to assess T-cell activation. PI3Kδ, PI3Kγ, phosphorylated protein kinase B (pAKT) and Signal Transducer and Activator of Transcription (STAT) protein expression were assessed by immunohistochemistry in bronchial biopsy tissue from asthma patients and healthy subjects. PI3Kδ expression in BAL CD3 cells was measured by flow cytometry.

**Results:**

JAK and PI3Kδ inhibitors reduced cytokine levels from both asthma and healthy BAL cells. Combining dexamethasone with either a JAK or PI3Kδ inhibitor showed an additive anti-inflammatory effect. JAK and PI3Kδ inhibitors were shown to have direct effects on T-cell activation. Immunohistochemistry showed increased numbers of PI3Kδ expressing cells in asthma bronchial tissue compared to controls. Asthma CD3 cells in BAL expressed higher levels of PI3Kδ protein compared to healthy cells.

**Conclusions:**

Targeting PI3Kδ or JAK may prove effective in reducing T-cell activation and the resulting cytokine production in asthma.

**Electronic supplementary material:**

The online version of this article (doi:10.1186/s12931-016-0436-2) contains supplementary material, which is available to authorized users.

## Background

Airway inflammation is a key feature of asthma. There are increased lymphocyte numbers in the lungs of patients with asthma, with many patients showing a T-helper 2 (TH2) response, associated with eosinophilic inflammation [1]. However, there is also evidence of an increased T-helper 1 (TH1) response in asthma, with IFNγ overproduction [2], and a role for T-helper 17 (TH17) cells in promoting neutrophilic inflammation in asthma [3].

Inhaled corticosteroids (ICS) are the most widely used anti-inflammatory treatments for asthma. However, many patients with moderate to severe asthma suffer with persistent symptoms despite the use of high doses of ICS [4]. Novel anti-inflammatory therapies are needed for asthma patients. Novel drugs that target lymphocyte cytokine production may be of therapeutic benefit in asthma.

Most pro-inflammatory cytokines signal through Janus Kinase (JAK) proteins following binding to cytokine receptors, resulting in activation of signal transducer and activator of transcription (STAT) proteins [5]. STATs act as transcription factors, and play a key role in lymphocyte differentiation and activation; IL-12 induces STAT4 activation promoting TH1 differentiation, IL-4 activates STAT6 promoting TH2 differentiation and IL-6, IL-21 and IL-23 all stimulate STAT3-dependent TH17 differentiation [6, 7].

Another intracellular kinase that controls lymphocyte function is PI3Kδ; this lipid kinase is involved in lymphocyte activation, proliferation and differentiation. T-cell receptor (TCR) stimulation results in activation of the PI3Kδ/pAKT pathway [8–10], and PI3Kδ inhibition reduces TCR stimulated cytokine production from healthy human peripheral blood lymphocytes [11].

Animal models have demonstrated roles for PI3Kδ and JAK/STAT signalling in airway inflammation [12–14]. Drugs that target PI3Kδ or JAK have the potential to suppress lymphocyte activation in asthma. We sought to provide validation of these novel drug targets by investigating their function and expression using lung cells and tissues from patients with asthma. The aims of this study were to evaluate the anti-inflammatory effects of PI3Kδ and JAK inhibition on release of T-cell derived cytokines from asthma bronchoalveolar lavage (BAL) cells, and to investigate activation of these pathways in asthma patients compared to healthy subjects by immunohistochemical analysis of PI3Kδ, PI3Kγ, pAKT and pSTAT proteins in bronchial biopsies. We also investigated the potential additive anti-inflammatory effects of a JAK or PI3Kδ inhibitor used with a corticosteroid on cytokine production from asthma lymphocytes, and the effects of these drugs on T-cell activation.

## Methods

### Patients

Twenty five moderate to severe asthma patients and 21 healthy subjects were recruited for bronchoscopy (see Table 1 for clinical characteristics). BAL cells were collected from 12 asthma patients and 11 healthy subjects for cytokine analysis, while biopsies were collected from 13 asthma and 12 healthy subjects; some patients provided both samples (8 asthma patients and 9 healthy subjects). BAL cells from 9 asthma patients and 6 healthy subjects were used for pSTAT5 and/or PI3Kδ analysis. All BAL cells were used for cell culture or flow cytometry; due to limited cell numbers, no cytospins for differential cells counts were collected. In order to estimate the percentage of lymphocytes present in BAL samples, we performed differential cell counts on BAL cytospins collected historically for research purposes. The clinical features of these 36 asthma patients and 15 healthy subjects are described in Additional file 1: Table S1. Blood was collected from a total of 5 asthma patients and 15 healthy subjects. These participants differed from those undergoing bronchoscopy (clinical features are summarised in Additional file 1: Table S1). Asthma patients were using ICS at a dose >800 μg/day beclomethasone equivalent, had an asthma control questionnaire (ACQ) score >1, and demonstrated >12 % FEV_1_ reversibility to salbutamol or a methacholine PD_20_ < 16 mg/ml. Healthy subjects had no history of chronic respiratory disease and had no evidence of airflow obstruction or a chest infection within 4 weeks prior to recruitment. All subjects were never smokers. Subjects were excluded if they had any history of non-asthmatic lung disease or an asthma exacerbation within 4 weeks prior to recruitment, as defined by a worsening in symptoms requiring a change in treatment and/or hospitalization. The study and sample collection was approved by the local research ethics committee (Bronchoscopy: NRES Committee North West – Greater Manchester South; REC Ref: 06/Q1403/156; Blood: North West Preston Research Ethics Committee, REC Ref: 10/H1016/25; Resected lung tissue for immunohistochemistry controls: South Manchester Research Ethics Committee, REC Ref: 03/SM/396) and all subjects provided written informed consent.

**Table 1:** Demographics, clinical characteristics and BAL cell yields for bronchoscopy subjects

	Healthy	Asthma
Number	21	25
Sex (m/f)	10/11	13/12*
Age	38.5+/− 13.1	43.8 +/−12.0*
Age of asthma diagnosis	N/A	16.1 +/−16.0
Body Mass Index	25.7 +/− 3.5	27.3 +/− 3.3*
Atopy (Y/N)	1/20	21/4^a^
ACQ score	N/A	1.85 +/− 0.79
FEV1 % predicted	103.7+/−17.2	72.4 +/− 15.5***
FEV1/FVC	78.8 +/−5.4	65.8 +/− 10.4***
Reversibility (%)	3.2 +/− 2.6	18.9 +/− 17.4**
SABA (y/n)	0/21	24/1^a^
LABA (y/n)	0/21	21/4^a^
ICS (y/n)	0/21	25/0**
Beclomethasone dose (μg/day)	0	1125+/− 558
BAL cell yields (×10^6^/ml BAL)	0.099 +/− 0.038	0.107 +/−0.072*

Data is presented as mean +/− standard deviation. Comparisons between groups were by T-test: *p > 0.05; **p < 0.01; ***p < 0.001, or by Chi-square test: ^a^ *p* < 0.001

*Abbreviations*: *ACQ* asthma control questionnaire, *FEV*_*1*_ Forced expiratory volume in one second, *FVC* forced vital capacity, *SABA* short acting β-agonist, *LABA* long acting β-agonist, *ICS* inhaled corticosteroid; *N/A* not applicable

### Cell collection and culture

The methods are fully described in the online Additional file 2: Supplementary Method. Briefly, BAL cells were treated with either a JAK inhibitor, tofacitinib (previously named CP-690550) [14], a PI3Kδ inhibitor, PIK-294, [15] (both synthesized in the Department of Medicinal Chemistry of Almirall R&D, Sant Feliu de Llobregat, Barcelona, Spain) or dexamethasone (Sigma-Aldrich, Poole, UK) for 1 h before addition of 5 ng/ml CD3 (ExBio, Vestec, Czech Republic product code: 12-631-C100) and 10 ng/ml CD28 (Biolegend, London, UK product code: 302913) antibodies to induce a T-cell receptor (TCR) specific response. Enzymatic profiles of tofacitinib [16] and PIK-294 [15] are shown in Additional file 3: Table S2. Cytotoxic effects of the drugs were assessed in TCR-stimulated PBMCs by Pierce LDH assay (Life Technologies, Paisley, UK) and propidium iodide flow cytometry assay (BD Bioscience, Oxford, UK); Additional file 4: Figure S1. When sufficient cells allowed, dexamethasone (1-10000nM) with 100nM of tofacitinib or PIK-294 were added before TCR stimulation; 100nM was chosen as it demonstrated submaximal inhibition of IFNγ from BAL cells from the first 3 patients. For PIK-294, 100nM also showed selectivity for PI3Kδ over other PI3K isoforms (see Additional file 3: Table S2). BAL cells were depleted of T-cells using the EasySep Human CD3 positive selection kit (Stemcell Technologies, Cambridge, UK); flow cytometry demonstrated that between 95.6 and 100 % of BAL CD3^+^ cells were removed.

### Cytokine analysis

IL-13, IFNγ, IL-17, TGF-β and IL-12 were measured in cell culture supernatants by “Ready-Set-Go!” ELISA (eBioscience, UK). Lower levels of quantification (LLOQ) for all assays were 4 pg/ml. IL-6 was measured by Duoset ELISA (R&D Systems, Abingdon, UK); LLOQ was 9.4 pg/ml.

### Flow cytometric analysis of pSTAT5 and PI3Kδ

Lymphocytes were isolated from blood using a Human T-cell isolation Kit (Stemcell Technologies, Cambridge, UK). Purity was assessed by flow cytometry, with a mean CD3^+^ count of 94.2 %. Purified T-cells were treated with 1000nM Dexamethasone, PIK-294 and tofacinib for 1 h before TCR-stimulation for 4 h. Cells were fixed, permeabilised, and labelled with CD3 and pSTAT5 antibodies. Analysis was carried out on a CANTO II flow cytometer (BD Biosciences, Oxford, UK). Full methods are in the online Additional file 2: Supplementary Method.

BAL cells were fixed, permeabilised, and labelled with CD3 and PI3Kδ antibodies. Analysis was carried out on a CANTO II flow cytometer (BD Biosciences, Oxford, UK). Full methods are in the online Additional file 2: Supplementary Method.

### Immunohistochemistry

Analysis of pSTATs 1, 3, 5 and 6, pAKT, PI3Kδ, PI3Kγ and CD3 were carried out by immunohistochemistry on bronchial biopsies, with analysts being blinded to subject classification. Full methods are in the online Additional file 2: Supplementary Method and Additional file 5: Table S3.

### Statistical analysis

Data distribution was determined by Kolmogorov-Smirnov test. Clinical characteristics were normally distributed and comparisons between groups by T-test or Chi-square test. Absolute IFNγ and IL-13 levels were normally distributed, while IL-17 was distributed non-parametrically. Intragroup analysis of drug effects was by ANOVA with Bonferroni post-hoc test for parametric data or Friedman test with Dunn’s post-hoc test for IL-17 data, and used absolute cytokine values. Percentage inhibition data was all parametrically distributed. Asthma vs healthy comparisons for the percentage inhibition effect of each drug on each cytokine were by 2-way ANOVA. Comparisons of the percentage inhibition effects for each drug between cytokines were by 2-way ANOVA with Tukey multiple comparison test. IC_35_ values, as well as IC_50_, are presented as >50 % inhibition was not always achieved for IL-17. The effects of dexamethasone with or without 100nM tofacitinib or PIK-294 were evaluated by 2-way ANOVA with Sidak post-hoc test. STAT5 activation and IL-6 levels were normally distributed; drug effects were analysed by 1-way ANOVA with a Dunnett’s multiple comparison test against the stimulated control. Comparisons of pAKT, PI3Kγ, pSTAT6 and CD3 PI3Kδ expression between healthy subjects and asthma patients were by T-test as the results were normally distributed, while other immunohistochemistry markers were compared using Mann-Whitney test as these were non-parametrically distributed. All statistical analysis was performed using Prism 6.04 (http://www.graphpad.com).

## Results

The demographics of the participants who underwent bronchoscopy are shown in Table 1. For asthma patients, the mean ACQ score was 1.85 and the mean FEV_1_ was 72.4 % predicted, indicating moderate to severe symptomatic asthma. The age, body mass index and BAL cell yields were similar between groups.

### TCR activation of BAL cells

TCR stimulation of BAL cells from asthma patients (*n* = 12) and healthy subjects (*n* = 11) was performed; IL-13 levels were higher in stimulated asthma compared to healthy cells (means 370 versus 130 pg/ml respectively; *p* = 0.02). There were no other differences between groups in either basal or stimulated conditions (Additional file 6: Table S4).

### Effects of inhibition of PI3Kδ and JAK on TCR responses in BAL cells

**Fig. 1:**
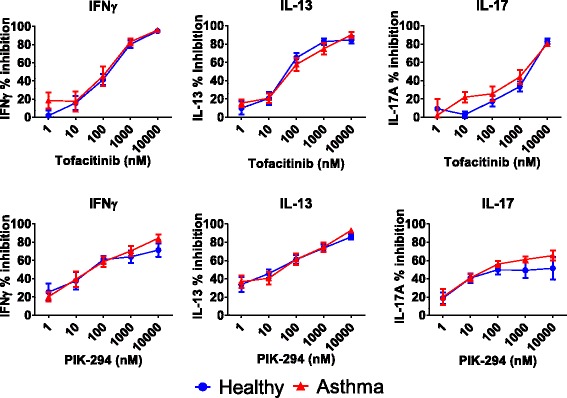
Effects of PIK-294 and tofacinib on cytokine release in BAL cells from asthma patients (*n* = 12) and healthy subjects (*n* = 11). BAL cells were treated with varying concentrations of PIK-294 or tofacitinib for 1 h before being stimulated with antibodies against CD3 and CD28 to induce a TCR-specific response. Cytokines were measured by ELISA. Data is presented as mean % inhibition (+/−SEM) of maximum cytokine release

Both JAK (tofacitinib) and PI3Kδ (PIK-294) inhibitors suppressed TCR stimulated IFNγ, IL-13 and IL-17 production (Fig. 1). The percentage inhibitions observed were similar in asthma patients compared to healthy subjects, with all 2-way ANOVA *p* values >0.05. The % inhibitions observed at the highest drug concentration (10 μM) ranged from 52 to 93 % for PIK-294, and 80 to 96 % for tofacitinib and did not differ between subject groups (Table 2). PIK-294 and tofacitinib IC_35_ and IC_50_ values are shown in Table 2; the values for IL-17 inhibition were generally higher compared to the other cytokines.

**Table 2:** Inhibition of cytokine production by dexamethasone, tofacitinib and PIK-294 in TCR-stimulated BAL cells from asthma patients and healthy subjects

Compound	Cytokine	IC_35_ values (nM)		IC_50_ values (nM)		Mean % inhibition at 10 μM +/− SD	
		Healthy	Asthma	Healthy	Asthma	Healthy	Asthma
Dexamethasone	IFNγ	2.6	3.4	7.6	28.7	75.2 +/− 20.8	69.1 +/− 26.7
	IL-13	<1.0^a^	<1.0^a^	1.1	1.2	96.6 +/− 6.8	77.3 +/− 31.1
	IL-17	8.7	267.8	1098	>10,000^b^	57.6 +/− 15.3	45.8 +/− 21.7
Tofacitinib	IFNγ	56.8	56.7	135.2	141.1	96.1 +/− 2.9	94.9 +/− 7.2
	IL-13	24.8	26.5	48.8	75.6	85.8 +/− 9.4	90.0 +/− 11.5
	IL-17	297	1042	1321	2495	80.4 +/− 9.2	83.1 +/− 11.0
PIK-294	IFNγ	6.8	5.3	31.7	35.8	77.5 +/− 17.1	84.4 +/− 14.5
	IL-13	1.3	<1.0^a^	19.0	31.2	87.4 +/− 9.3	93.0 +/− 5.5
	IL-17	9.6	10.1	368.4	75.9	61.3 +/− 14.7	70.3 +/− 12.2

Absolute IC_35_ and IC_50_ values calculated from the mean % inhibition for each drug concentration tested. ^a^IC_35_ value uncalculatable as >35 % mean inhibition observed with 1.0 nM of drug; ^b^IC_50_ value uncalculatable as <50 % mean inhibition observed with 10,000 nM of drug

Toxicity tests showed that PIK-294 and tofacitinib had no cytotoxic effects on TCR-stimulated PBMCs at all concentrations tested (Additional file 4: Figure S1).

### Combination effects of dexamethasone and JAK or PI3Kδ inhibitors on TCR responses

**Fig. 2:**
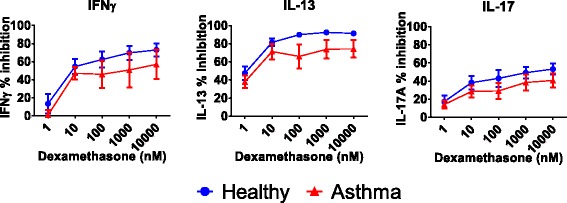
Effect of dexamethasone on cytokine release in BAL cells from asthma patients (*n* = 12) and healthy subjects (*n* = 11). BAL cells were treated with varying concentrations of dexamethasone for 1 h before being stimulated with antibodies against CD3 and CD28 to induce a TCR-specific response. Cytokines were measured by ELISA. Data is presented as mean % inhibition (+/− SEM) of maximum cytokine release

The mean inhibitory effect of dexamethasone on all three cytokines was numerically greater in cells from controls compared to those from asthma patients, but these differences were not statistically significant (Fig. 2 and Table 2). Dexamethasone IC_35_ and IC_50_ values for IL-17 and IFNγ (Table 2) were lower in healthy subjects compared to asthma. The effect of dexamethasone varied between cytokines, with IL-17 being inhibited less than IL-13 and IFNγ (ANOVA analysis in healthy subjects and asthma; *p* = 0.0002 and *p* = 0.0006 respectively; Additional file 7: Figure S2). A notable difference was that dexamethasone was more potent than PIK-294 and tofacinib for inhibition of IL-13 from both asthma and healthy subjects, with at least 10-fold lower IC_50_ values.

**Fig. 3:**
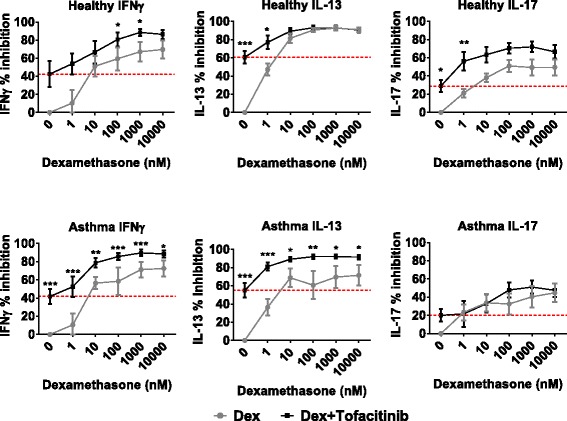
Additive effects of dexamethasone with tofacinib on cytokine release in BAL cells from asthma patients (*n* = 10) and healthy subjects (*n* = 8). BAL cells were treated with varying concentrations of dexamethasone with or without 100nM of tofacitinib for 1 h before being stimulated with antibodies against CD3 and CD28 to induce a TCR-specific response. Cytokines were measured by ELISA. Data is presented as mean % inhibition (+/−SEM) of maximum cytokine release. Red line denotes inhibition by 100nM tofacinib alone. Comparisons between with or without tofacitinib were analysed by 2-way ANOVA with Sidak post-test using cytokine levels: *p < 0.05; **p < 0.01; ***p < 0.001

**Fig. 4:**
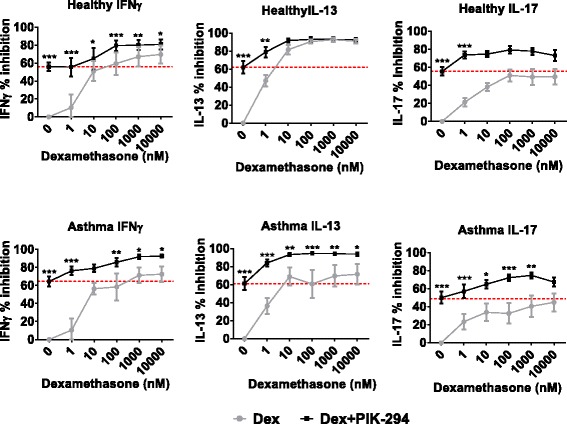
Additive effects of dexamethasone with PIK-294 on cytokine release in BAL cells from asthma patients (*n* = 10) and healthy subjects (*n* = 8). BAL cells were treated with varying concentrations of dexamethasone with or without 100nM of PIK-294 for 1 h before being stimulated with antibodies against CD3 and CD28 to induce a TCR-specific response. Cytokines were measured by ELISA. Data is presented as mean % inhibition (+/−SEM) of maximum cytokine release. Red line denotes inhibition by 100nM PIK-294 alone. Comparisons between with or without PIK-294 were analysed by 2-way ANOVA with Sidak post-test using cytokine levels: *p < 0.05; **p < 0.01; ***p < 0.001

There were sufficient BAL cells from 10 asthma patients, and 8 healthy subjects to perform combination experiments; cells were treated with increasing concentrations of dexamethasone plus 100nM of either tofacitinib (Fig. 3) or PIK-294 (Fig. 4); this concentration for tofacitinib and PIK-294 was chosen on the basis of causing submaximal inhibition, and for PIK-294, showing specificity for PI3Kδ over other isoforms (see Fig. 1 and Additional file 3: Table S2).

Tofacitinib plus dexamethasone produced a greater effect than dexamethasone alone, with an additive effect on the inhibition of IL-13 and IFNγ production from asthma and healthy cells; this additive effect on IL-13 from healthy cells was only apparent at low dexamethasone concentrations (Fig. 3). Tofacitinib caused an additive effect on the suppression of IL-17 production from healthy cells, but this was not seen with cells from asthma patients. It should be noted that the tofacitinib concentration used (100nM) had little effect on IL-17 production from asthma cells when used without dexamethasone.

PIK-294 and dexamethasone combined caused additive suppression of all three cytokines measured from asthma and healthy cells; this additive effect on IL-13 from healthy cells was only apparent at low dexamethasone concentrations (Fig. 4).

### Lymphocyte specific effects of dexamethasone, PIK-294 and tofacinib

Historically collected samples showed that lymphocytes typically account for only 2 % of BAL cells in asthma patients and healthy subjects (Additional file 8: Table S5). The BAL cytokine work described above used a T-cell specific stimulant to initiate inflammatory responses in a mixed cell population. In this model, stimulated lymphocytes may activate other cell types, such as macrophages, which may be required for full lymphocyte activity. It is therefore possible that the inhibitory effects of the drugs on cytokine release were due to activity on non T-cells.

To demonstrate that TCR-induced cytokine release from BAL cells is T-cell dependent, BAL (*n* = 3), was depleted of CD3^+^ cells and the remaining cells treated with antibodies against CD3 and CD28 for 72 h; the resulting levels of IFNγ, IL-13 and IL-17 were all undetectable (data not shown). Attempts were made to isolate purified lymphocytes from BAL in order to investigate cytokine production after TCR stimulation. However, due to the low lymphocyte and high macrophage composition of the BAL (approximately 2 and >80 % respectively), a maximum lymphocyte purity of only 50 % was achievable. We therefore studied the effects of dexamethasone, PIK-294 and tofacinib on cytokine release from T-cells purified from the blood of asthma patients (*n* = 5). TCR-stimulation of isolated lymphocytes did not induce IFNγ, IL-13 or IL-17 (data not shown); suggesting that cross-talk between T-cells and other cells such as monocytes or macrophages is required to enable TCR-induced cytokine production.

**Fig. 5:**
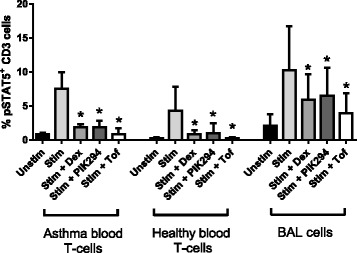
Effects of dexamethasone, PIK-294 and tofacinib on TCR-induced pSTAT5 activation in lymphocytes. Isolated blood T-cells from asthma patients (*n* = 5) and healthy subjects (*n* = 6) and BAL cells (*n* = 4 asthma plus *n* = 3 healthy) were treated with 1000nM dexamethasone, PIK-294 or tofacinib for 1 h before TCR-stimulation for 4 h. Levels of phosphorylated STAT5 in CD3^+^ cells were quantified by flow cytometry. Data is presented as mean % pSTAT5 positive CD3 cells +/− standard deviation. Drug effects were assessed by 1-way ANOVA with a Dunnett’s multiple comparison test against the stimulated no drug control: *p < 0.05

To demonstrate that the drugs had a direct effect on lymphocytes, TCR-induced STAT5 phosphorylation in purified T-cells, from the blood of asthma patients (*n* = 5) and healthy subjects (*n* = 6), was examined (Fig. 5). STAT5 is activated following TCR-induced production of IL-2, and is an early stage marker of T-cell proliferation [17]. The levels of STAT5 activation in T-cells were similar for both groups. Tofacinib (1 μM), inhibited STAT5 phosphorylation by 90.4 and 88.0 % in asthma and healthy cells respectively, while dexamethasone (1 μM) reduced activation by 74.2 % (asthma) and 76.0 % (healthy) and PIK-294 (1 μM) by 70.3 % (asthma) and 78.2 % (healthy). Inhibition of pSTAT5 in CD3 cells was also seen following TCR-stimulation of mixed BAL cells (Fig. 5).

TCR-stimulated BAL cells, from asthma patients (*n* = 5), were used to investigate whether the drugs had any effect on cytokines from other cell types, which could subsequently influence lymphocyte activity. We measured levels of IL-12, which stimulates IFNγ production in Th1 cells, and IL-6 and TGF-β, which govern IL-17 production in Th17 cells. IL-12, IL-6 and TGF-β are all produced by macrophage and other antigen presenting cells found in BAL [18–20]. IL-6 levels were not increased following TCR-stimulation of BAL cells, and release was not inhibited by dexamethasone, PIK-294 or tofacinib (Additional file 9: Figure S3). IL-12 and TGF-β levels were undetectable (data not shown).

### Expression of PI3K, pAKT and pSTAT proteins in bronchial biopsies from asthma patients and healthy subjects

Expression of pSTAT1, 3, 5 and 6, as well as pAKT, PI3Kδ and PI3Kγ, were assessed by immunohistochemistry in bronchial biopsies from healthy subjects (*n* = 12) and asthma patients (*n* = 13). Expression of all 7 proteins was detectable in airway epithelial cells and in infiltrating inflammatory cells, within the subepithelium, for both groups (see Additional file 10: Figure S4). Airway smooth muscle cells displayed clear nuclear expression of PI3Kδ and diffused cytoplasmic expression of pSTAT5 and pSTAT6 only.

**Fig. 6:**
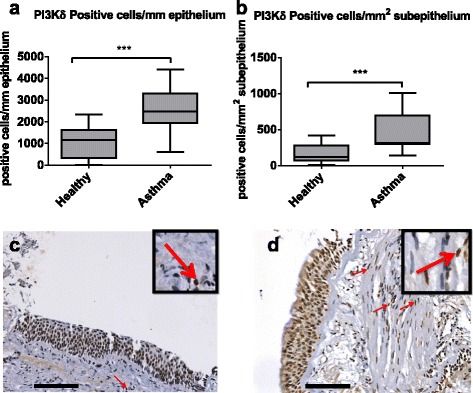
Expression of PI3Kδ in bronchial biopsies from asthma patients (*n* = 13) and healthy subjects (*n* = 12). PI3Kδ expression was examined in bronchial tissue by immunohistochemistry. Results are presented as positive cells per mm of epithelium (**a**) and positive cells per mm^2^ of subepithelium (**b**). Expression in asthma tissue was compared with healthy by Mann-Whitney tests. *p < 0.05; **p < 0.01; ***p < 0.001. Representative images show PI3Kδ expression in bronchial tissue from healthy subjects (**c**) and asthma patients (**d**). *Arrows* and amplified images highlight positive staining in the subepithelial inflammatory cells. *Black bars* represent 100 μm

The number of cells expressing PI3Kδ was higher in asthma patients compared to healthy subjects in both the epithelium (median expression 747 cells/mm vs 314 cells/mm *p* = 0.0005) and subepithelial areas (median expression 314 cells/mm^2^ vs 119 cells/mm^2^ *p* = 0.0012) (Fig. 6). No differences were found between groups for any of the other proteins measured (*p* > 0.05 for all comparisons) (Additional file 11: Table S6).

**Fig. 7:**
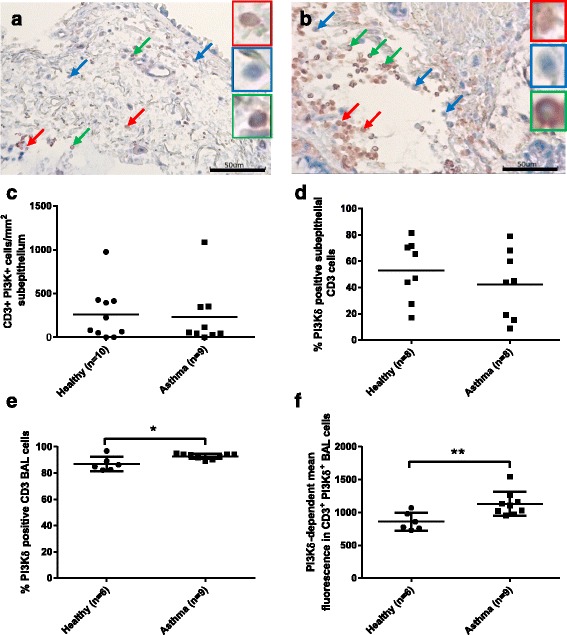
Expression of PI3Kδ in subepithelial and BAL T-cells from asthma patients and healthy subjects. PI3Kδ expression was examined in CD3 cells in subepithelial bronchial tissue and BAL by immunohistochemistry and flow cytometry, respectively. Representative images show PI3Kδ expression in bronchial tissue from healthy subjects (**a**) and asthma patients (**b**). Arrows and amplified images highlight example staining: Red for CD3 only; Blue for PI3Kδ only; Green for dual CD3 and PI3Kδ. Black scale bars represent 50 μm. Data is presented as (**c**) numbers of CD3^+^ PI3Kδ^+^ cells per mm^2^ subepithelium (Healthy *n* = 10; Asthma *n* = 9); (**d**) percentage of subepithelial CD3 cells expressing PI3Kδ (Healthy *n* = 8; Asthma *n* = 8); (**e**) percentage of BAL CD3 cells expressing PI3Kδ (Healthy *n* = 6; Asthma n = 9) and (**f**) PI3Kδ-dependent mean fluorescence intensity in dual labelled CD3^+^ PI3Kδ^+^ BAL cells (Healthy *n* = 6; Asthma *n* = 9), with bar illustrating mean values. The percentage of subepithelial CD3 cells expressing PI3Kδ could not be calculated for *n* = 2 healthy and *n* = 1 asthma, due to lack of CD3 cells. Differences between healthy and asthma were assessed by T-test: *p < 0.05; **p < 0.01

To further assess PI3Kδ expression in subepithelial lymphocytes, dual label immunohistochemistry was carried out on bronchial biopsies for CD3 and PI3Kδ (Fig. 7a-d); the percentage of CD3 cells expressing PI3Kδ and the absolute numbers of CD3/PI3Kδ dual positive cells were similar in asthma and healthy samples. Sample numbers (asthma *n* = 9, healthy *n* = 10) are lower than in Fig. 6, due to insufficient tissue from some donors.

For participants who donated BAL for cytokine release experiments and biopsies for CD3 analysis (*n* = 7 asthma; *n* = 7 healthy), comparisons were made between cytokine release and PI3Kδ expression. Neither the number of PI3Kδ-positive CD3 cells, nor the percentage of CD3 cells expressing PI3Kδ, correlated with the levels of TCR-stimulated IFNγ, IL-13 or IL-17 released (*p* > 0.05 for all comparisons).

PI3Kδ expression was also examined in BAL CD3 cells by flow cytometry; these samples differed from those used for cytokine analysis. There was a small, but significant, increase in the percentage of BAL CD3 cells expressing PI3Kδ in asthma (*n* = 9) samples, compared to healthy samples (*n* = 6), 92.8+/−2.0 % and 87.0+/−5.4 % respectively (Fig. 7e; *p* = 0.01). Analysis of PI3Kδ-specific fluorescence in PI3Kδ positive CD3 cells suggests that T-cells from asthma patients express higher levels of PI3Kδ protein than cells from healthy individuals (*p* = 0.009) Fig. 7f).

## Discussion

Inhibition of JAK or PI3Kδ suppressed cytokine production from BAL cells of patients with moderate to severe asthma and reduced T-cell activation. Furthermore, there were additive anti-inflammatory effects when JAK and PI3Kδ inhibitors were used in combination with corticosteroids. Numbers of PI3Kδ expressing cells were increased in the airways of asthma patients compared to controls, as well as levels of PI3Kδ protein in BAL CD3 cells, indicating increased activity of this signalling pathway. Overall, these findings indicate considerable potential to use PI3Kδ and JAK inhibitors to target T-cell associated inflammation in asthma. The major findings and limitations of the study are now discussed.

In this study, we used a mixed BAL cell model to assess the anti-inflammatory effects of PIK-294 and tofacinib. We believe that this is a more physiologically relevant model than using isolated T-cells for the following reasons: firstly, anti-inflammatory treatments for asthma, such as corticosteroids, and potentially JAK and PI3Kδ inhibitors, do not target specific cell types, such as lymphocytes, but have wide ranging effects on different inflammatory cells. Secondly, cross-talk between various cells types influences the inflammatory response, and these effects cannot be observed in single cell type models.

### PI3Kδ inhibition

PI3Kδ is known to be involved in TCR-associated lymphocyte activation. We showed that PI3Kδ inhibition suppresses IFNγ, IL-13 and IL-17 release from BAL cells. Using peripheral blood T-cells, it has been shown that a PI3Kδ selective inhibitor inhibits TCR stimulated IFNγ and IL-17 production from healthy subjects, and IL-5 and IL-13 production from patients with allergy [11]. Furthermore, the TH1 and TH17 responses of T-cells from the synovial fluid of rheumatoid arthritis patients were also suppressed by PI3Kδ selective inhibition [11]. We now show that PI3Kδ selective inhibition also has a broad range of effects on TH1, TH2 and TH17 responses in lung cells from healthy subjects and asthma patients.

We demonstrated that PIK-294 inhibited TCR-specific activation of lymphocytes isolated from PBMCs by measuring STAT5 phosphorylation in lymphocytes. However, CD3/CD28 stimulation did not result in upregulation of cytokine production in isolated lymphocytes, suggesting that other cell types, such as monocytes/macrophages, are required to facilitate lymphocyte cytokine production after TCR stimulation. Previous work has shown that PI3Kδ is involved in the activation of monocytes/macrophage following direct contact with stimulated T-cells [21], highlighting the interaction between these cell types in inflammatory responses. However, we showed that PIK-294 had no effect on cytokines that are known to influence T cell responses, and conclude that the effect of this PI3Kδ inhibitor on IFNγ, IL-13 and IL-17 production in the supernatant of TCR-stimulated BAL cells is most likely through direct effects on lymphocytes.

PI3Kδ expression was elevated in the epithelium and sub-epithelium of bronchial biopsies of asthma patients compared to healthy subjects. There is evidence to support increased expression of PI3Kδ in COPD alveolar macrophages [22] and COPD blood neutrophils [23] compared to controls, but to our knowledge there are no similar data in asthma. PI3Kγ expression was not elevated in asthma tissue compared to controls, suggesting that PI3Kδ is the dominant isoform that is upregulated in asthma. The numbers of subepithelial CD3 cells expressing PI3Kδ did not differ between asthma and healthy tissue, suggesting that the elevation of PI3Kδ in asthma tissue is due to an increase in other cell types which express PI3Kδ, such as neutrophils or macrophages. In BAL, however, the percentage of CD3 cells expressing PI3Kδ was slightly increased in asthma compared to healthy samples, and, potentially more interestingly, CD3^+^ PI3Kδ^+^ BAL cells from asthma patients expressed higher levels of PI3Kδ protein than cells from healthy subjects. Increased PI3Kδ activity is the cause of PI3Kδ syndrome, which is characterised by repeat respiratory infections and progressive airway damage [24]. Potentially, the occurrence of similar characteristics in more severe asthma patients may be linked to increased PI3Kδ levels demonstrated in our study; this requires further investigation.

Interestingly, PI3Kδ expression was increased in epithelial cells, as well as infiltrating inflammatory cells, from asthma patients. We set out *a priori* to investigate the effects of PI3Kδ inhibition on BAL cells, and collected cell culture samples specifically for this purpose. We did not collect epithelial cells for culture, but our positive findings for this cell type indicate that further studies with bronchial epithelial cells are required to explore the function of PI3Kδ in asthma patients.

### JAK inhibition

TCR-stimulated release of IFNγ, IL-13 and IL-17 from BAL cells was JAK-dependent. IL-2 is a key cytokine involved in lymphocyte activation and proliferation, and signals through the IL-2 receptor/JAK/STAT5 pathway. We demonstrated that tofacitinib inhibited this IL-2 signalling pathway in T-cells isolated from PBMCs and CD3^+^ BAL cells, supporting a direct pharmacological effect of this drug on lymphocytes causing cytokine suppression in BAL cells. There was notable suppression of the TH1 and TH2 responses, with over 80 % inhibition of IFNγ and IL-13 secretion. Inhibiting TH1 responses may be therapeutically important in asthma; Raundhal et al. [2] recently demonstrated that BAL from severe asthma patients contains more IFNγ producing lymphocytes, than BAL from mild asthma patients. JAK inhibitors could potentially be used to target this component of the immune response in severe asthma.

The effects of tofacitinib on IL-17 production at concentrations of 1 μM and below were modest, although there was a sharp rise in efficacy at 10 μM. This indicates low potency for this drug on IL-17 production, confirmed by the high IC_35_ values >0.25 μM; this contrasts sharply with low nanomolar tofacitinib IC_35_ values for other cytokines (see Table 2), and suggests that JAK inhibitors may not reduce IL-17-driven responses in asthma.

There was no difference in activated STAT1, 3, 5 and 6 levels in bronchial biopsies from asthma patients compared to controls. Previous studies involving lung tissue from asthma patients have also failed to find elevated levels of pSTAT1 [25], although Sampath et al. [26] reported increased expression in steroid naïve asthma compared to controls. It is possible that high ICS doses used by patients in our study inhibited STAT1 activity. Rhinovirus infection of epithelial cells increases STAT1 expression [27], and increases in STAT1 activity may be more prominent during viral induced exacerbations of asthma.

Ruwanpura et al. [28] showed that pSTAT3 expression in COPD lung tissue is related to the degree of airway inflammation; perhaps our negative findings are indicative of low grade inflammation in the patients sampled. It has been reported that total STAT6 expression is increased in the bronchial epithelium of asthma patients compared to controls [29, 30], but others could not reconfirm this result [31]. Interestingly, inhaled allergen challenge in asthma patients induces pSTAT6 expression in epithelial cells [32], demonstrating dynamic regulation of STAT-associated activity with airway inflammation. Our study focused on phosphorylated STAT6, and found no difference between groups. Overall, our results suggest that the dynamic regulation of STAT protein phosphorylation interferes with the ability to detect STAT dysregulation by immunohistochemistry in this context.

### Combination therapy

We have previously shown that T-cell receptor stimulation of BAL cells causes IL-13 production that is less sensitive to corticosteroids in asthma patients compared to controls [33]. The current study showed numerical trends for reduced corticosteroid effects in asthma, but these did not reach statistical significance, perhaps due to variation in a limited sample size. In our study, IL-13 production was greater in asthma cells compared to controls. Potentially, this additional production is induced by corticosteroid insensitive mechanisms, which caused reduced efficacy of dexamethasone in our experiments. Unlike corticosteroid sensitive patients, systemic prednisolone treatment does not reduce IL-13 production in corticosteroid resistant asthma [34]. The low nanomolar IC_35_ values of dexamethasone for IL-13 in asthma patients should be noted, but the maximal efficacy at the higher concentrations was only approximately 75 % inhibition, compared to over 90 % in healthy samples. IL-13 is involved in allergic responses, fibrosis and mucus hypersecretion [35]. Our results suggest that corticosteroid use in clinical practice is unlikely to completely inhibit IL-13 production from lymphocytes, even at high doses.

Corticosteroid suppression of IL-17 was lower compared to other cytokines. This variation in corticosteroid effects on different cytokines has been observed in other cell types [19, 36], and may be due to differences in the signaling pathways that control cytokine gene expression. Recent studies have shown that CD4 cells from steroid insensitive asthma patients release more IL-17 than cells from steroid sensitive patients [37] and that, unlike TH2 cells, IL-17 producing BAL T-cells from severe asthma patients are insensitive to corticosteroid-induced cell death [38]. The clinical implication of these findings is that TH17 inflammation in asthma is relatively corticosteroid insensitive.

There were increased anti-inflammatory effects when combining corticosteroid with either tofacitinib or PIK-294, compared to either drug alone; these additive effects may be useful in clinical practice, as drugs of these classes may be used in combination with corticosteroids. Due to cell number limitations we were unable to perform analysis using multiple concentrations of both dexamethasone and kinase inhibitors to determine whether these combination effects are truly additive or synergistic.

Previous studies have shown that PI3Kδ inhibition can enhance the effects of corticosteroids; this has been observed using COPD peripheral blood monocytes and monocytic cell lines exposed to oxidative stress [22]. The current data shows an additive effect of these two classes of drugs in asthma lymphocytes.

Previous studies using other immune cells types have also shown that JAK inhibitors combine with corticosteroids to provide enhanced anti-inflammatory effects [25, 39]. Tofacitinib had low potency on IL-17 production, and consequently did not have an additive effect on IL-17 production when combined at 100nM concentration with dexamethasone. In contrast, PI3Kδ inhibition combined with dexamethasone to provide additive anti-inflammatory effects on all cytokines including IL-17, suggesting promise for this drug combination for suppressing IL-17 production in asthma.

## Conclusion

Inhibition of either JAK or PI3Kδ signalling suppresses cytokine production from asthma and healthy BAL cells, and reduces T-cell activation. We provide evidence of additive effects when these drugs are combined with corticosteroids. These results promote the development of these drug classes for the treatment asthma. The upregulation of PI3Kδ expression in the airways of asthma patients further underscores the potential of targeting this kinase to suppress inflammation in this disease.

## Additional files

Additional file 1: Table S1. Demographics and clinical features of blood donating subjects and donors of historically collected BAL cytospins. (DOC 33 kb)
Additional file 2: Supplementary Method. (DOC 47 kb)
Additional file 3: Table S2. In vitro enzymatic profiles for tofacitinib and PIK-294. (DOC 35 kb)
Additional file 4: Figure S1. Cytotoxic effects of PIK-294, tofacinib and dexamethasone. PBMCs from healthy subjects (*n* = 3) were treated with PIK-294, tofacinib or dexamethasone for 1 h prior to TCR-stimulation for 72 h. Cytotoxicity was assessed by LDH assay (A) or propidium iodide flow cytometry assay (B). Cytotoxic effects were assessed by 1-way ANOVA with Dunnett’s post-hoc test against the no drug DMSO control. All non-significant. (PPTX 211 kb)
Additional file 5: Table S3. Immunohistochemistry retrieval buffers and antibody dilutions. (DOC 34 kb)
Additional file 6: Table S4. Baseline and TCR-stimulated Cytokine levels in BAL cells. (DOC 35 kb)
Additional file 7: Figure S2. Comparison of % inhibition of IFNγ, IL-13 and IL-17 for dexamethasone, tofacitinib and PIK-294. BAL cells from asthma patients (*n* = 12) and healthy subjects (*n* = 11) were treated with dexamethasone, tafacitinib or PIK-294 for 1 h before being stimulated with antibodies against CD3 and CD28 to induce a TCR-specific response. Cytokines were measured by ELISA and data is presented as the mean (+/− SEM) % inhibition in relation to the stimulated no drug control. (PPTX 742 kb)
Additional file 8: Table S5. BAL Differential Cell Counts from historically collected samples. (DOC 29 kb)
Additional file 9: Figure S3. Effects of Dexamethasone, PIK-294 and tofacinib on IL-6 from TCR-stimulated BAL cells. BAL cells from asthma patients (*n* = 5) were treated with varying concentrations of dexamethasone with or without 100nM of tofacitinib for 1 h before being stimulated with antibodies against CD3 and CD28 to induce a TCR-specific response. IL-6 was measured by ELISA. Data is presented as mean +/− standard deviation. Drug effects were analysed by 1-way ANOVA: *p* = 0.22. (PPTX 89 kb)
Additional file 10: Figure S4. Representative images for Immunohistochemistry negative controls. Bronchial biopsy tissue was stained using primary antibodies against PI3Kδ, PI3Kγ, pAKT, STAT1, STAT3, STAT5 and STAT6. Sequential sections were used for negative controls using either an isotype control negative antibody at the same concentration as the various primary antibodies, or with the omission of the primary antibody. Negative controls for each primary antibody were assessed in sequential tissue sections from *n* = 3 patients. Black bar represents 100 μm. (PPTX 487 kb)
Additional file 11: Table S6. Expression of pSTATs, pAKT, PI3Kγ and PI3Kγ by immunohistochemistry in bronchial biopsies. (DOC 41 kb)

## Abbreviations

ACQ: Asthma control questionnaire
BAL: Bronchoalveolar lavage
FEV_1_: Forced expiratory volume in 1 second
FVC: Forced vital capacity
GR: Glucocorticoid receptor
IC: Inhibitory concentration
ICS: Inhaled corticosteroids
IFNγ: Interferon gamma
IL: Interleukin
JAK: Janus-activated kinases
LABA: Long acting β-agonist
N/A: Not applicable
pAKT: Phophorylated protein kinase B
PBMC: Peripheral blood mononuclear cells
PD_20_: Provocative Dose to induce 20 % fall in FEV_1_
PI3Kγ: Phosphatidylinositol 3-kinase gamma
PI3Kδ: Phosphatidylinositol 3-kinase delta
SABA: Short acting β-agonist
SD: Standard deviation
SOCS: Suppressor of cytokine signalling
STAT: Signal transducer and activator of transcription
TCR: T-cell receptor
TH1: T-helper 1
TH17: T-helper 17
TH2: T-helper 2
