# Supplementary material for: Anti-inflammatory potential of PI3Kδ and JAK inhibitors in asthma patients
Source: Respir Res. 2016 Oct 4;17:124. doi: 10.1186/s12931-016-0436-2 (PMC5051065; doi:10.1186/s12931-016-0436-2)
Supplement: Additional file 1: Table S1. — Demographics and clinical features of blood donating subjects and donors of historically collected BAL cytospins. (DOC 33 kb) [file 12931_2016_436_MOESM1_ESM.doc]

Supplementary Table 1: Demographics and clinical features of blood donating subjects and donors of historically collected BAL cytospins.

|  | Blood | | BAL Cytospins | |
| --- | --- | --- | --- | --- |
|  | Healthy | Asthma | Healthy | Asthma |
| Number | 15 | 5 | 15 | 36 |
| Sex (m/f) | 7/8 | 2/3^NS^ | 8/7 | 22/14^NS^ |
| Age | 32.3 +/- 10.7 | 54 +/- 18.4 | 44.7 +/- 9.0 | 42.3 +/- 10.1^ns^ |
| Atopy (Y/N) | 3/12 | 3/2^NS^ | 0/15 | 34/2^###^ |
| ACQ score | N/A | 1.9 +/- 1.0 | N/A | 1.3 +/- 0.7 |
| FEV1 % predicted | 100.7 +/- 11.4 | 63.0 +/- 9.0** | 96.5 +/- 10.5 | 84.9 +/- 17.2^ns^ |
| FEV1/FVC | 79.4 +/- 7.8 | 59.4 +/- 5.7** | 76.0 +/- 3.7 | 66.8 +/- 9.1* |
| Reversibility (%) | 4.45 +/- 5.6 | 20.2 +/- 20.3** | 2.7 +/- 3.4 | 12.3 +/- 1.2** |
| SABA (y/n) | 0/15 | 5/0^###^ | 0/15 | 36/0^###^ |
| ICS (y/n) | 0/15 | 5/0^###^ | 0/15 | 36/0^###^ |

Data is presented as mean +/- standard deviation. Comparison of age, Body Mass Index and BAL cell yields between healthy vs. asthma was by unpaired T-test; all p>0.05. Abbreviations: DCC: differential cell counts; ACQ: asthma control questionnaire; FEV_1_: Forced expiratory volume in one second; FVC: Forced vital capacity; SABA: Short acting β-agonist; ICS: Inhaled corticosteroid; N/A: not applicable. Comparisons between groups were by T-test: ^ns^p>0.05; **p<0.01; ***p<0.001, or by Chi-square test: ^ns^p>0.05; ^###^p<0.001
