# Supplementary material for: Anti-inflammatory potential of PI3Kδ and JAK inhibitors in asthma patients
Source: Respir Res. 2016 Oct 4;17:124. doi: 10.1186/s12931-016-0436-2 (PMC5051065; doi:10.1186/s12931-016-0436-2)
Supplement: Additional file 2: — Supplementary Method. (DOC 47 kb) [file 12931_2016_436_MOESM2_ESM.doc]

**SUPPLEMENTARY Method**

**METHODS**

**Cell Collection:**

BAL was collected from right and left upper lobes, with a maximum of 4 x 60ml of pre-warmed 0.9% sterile saline being administered to each lobe. The BAL was filtered (100μm filter, Becton Dickenson) and centrifuged (400g, 10 min at 4°C). The cell pellet was resuspended in RPMI 1640 medium supplemented with 10% v/v fetal calf serum, with 2 mM L-glutamine, 100 U/ml penicillin, and 100 µg/ml streptomycin. Viable counts were performed using trypan blue and cell concentration adjusted to 1x10^6^/ml.

**Cell culture:**

BAL cells were seeded at 1x10^5^ cells/well in 96-well plates and treated with either a JAK inhibitor, tofacitinib, a PI3Kδ inhibitor, PIK-294, both synthesized in the Department of Medicinal Chemistry of Almirall R&D (Sant Feliu de Llobregat, Barcelona, Spain) and/or dexamethasone (Sigma-Aldrich, Poole, UK) for 1 hr before addition of 5ng/ml CD3 (ExBio product code: 12-631-C100) and 10ng/ml CD28 (Biolegend product code: 302913) antibodies to induce a T-cell receptor (TCR) specific response. Cells were then left for 72 hrs before being centrifuged and supernatants collected and frozen at -20°C for subsequent cytokine analysis.

When there were sufficient cells, varying concentrations of dexamethasone with or without 100nM of the tofacitinib or PIK-294 were added before TCR stimulation for 72 hours. The concentration of 100nM was chosen as it demonstrated submaximal inhibition of IFNγ from BAL cells from the first 3 patients involved in the study.

**Flow cytometric analysis of pSTAT5:**

Lymphocytes were purified from Ficoll-Paque (GE Healthcare Life Sciences, Buckinghamshire, UK) prepared PBMCs using the Human T-cell isolation Kit (Stemcell Technologies, Cambridge, UK). Purified T-cells or BAL cells were treated with 1000nM Dexamethasone, PIK-294 and tofacinib for 1 hr before TCR-stimulation for 4 hrs. Cells were washed twice in FACS stain buffer (PBS with 2% foetal calf serum, 270g, 6min), fixed with Cytofix (BD Biosciences, Oxford, UK) for 10 min at 37^o^C, washed twice as before and permeabilised with Perm Buffer III (BD Biosciences, Oxford, UK) on ice for 30 minutes. Cells were washed twice more before being stained with CD3 APC-labelled and pSTAT5 Alexa Fluor 488 labelled antibodies (both BD Biosciences, Oxford, UK) for 30 min at room temperature in the dark. Samples were analysed on a Canto II flow cytometer using Diva 6.0 software. Auto-compensation was performed using purified T-cells stained with CD3 antibodies labelled with APC and Alexa Fluor 488 (BD Biosciences, Oxford, UK). Cells stained with CD3 APC and Alexa Fluor 488 isotype control antibodies were used as the fluorescence minus one (FMO) control to identify positive pSTAT5 staining in CD3 cells.

**Flow cytometric analysis of PI3Kδ:**

BAL cells were washed twice in FACS stain buffer (as above), fixed with Cytofix (BD Biosciences, Oxford, UK) for 10 min at 37^o^C, washed twice as before and permeabilised with Perm Buffer III (BD Biosciences, Oxford, UK) on ice for 30 minutes. Cells were washed twice more before being stained with CD3 APC-labelled (BD Biosciences, Oxford, UK) and PI3Kδ Alexa Fluor 488 labelled (antibodies-online.com, Germany) antibodies for 30 min at room temperature in the dark. Samples were analysed on a Canto II flow cytometer using Diva 6.0 software. Auto-compensation was performed using BAL cells stained with CD3 antibodies labelled with APC and Alexa Fluor 488 (BD Biosciences, Oxford, UK). Cells stained with CD3 APC and Alexa Fluor 488 isotype control antibodies were used as the fluorescence minus one (FMO) control to identify positive PI3Kδ staining in CD3 cells.

**Immunohistochemistry:**

For each patient, formalin-fixed, paraffin-embedded tissue biopsies were stained for immunohistochemical analysis. The primary antibodies used were as follows: pSTAT1 (Cat No: 9167, Cell Signalling, Danvers, USA), pSTAT3 (Cat No: 9145, Cell Signalling), pSTAT5 (Cat No: 9359, Cell Signalling), pSTAT6 (Cat No: ab28829, Abcam, Cambridge, UK), pAkt (Cat no: 9271, Cell Signalling), PI3Kγ (Cat No: 4252, Cell Signalling) and PI3Kδ (Cat No: Ab1678, Abcam).

Airway tissues were cut into 4μm sections and lifted onto a polysine coated glass slide. Following heat induced epitope retrieval for 20 min at 800 W (details of retrieval buffers used and antibody dilutions provided in Supplementary Table 3) primary antibodies, diluted in 1.5% normal serum (Vector Labs, Peterborough, UK) 0.05% triton X-100 (Sigma-Aldrich, Poole, UK), were applied overnight at 4°C. Endogenous peroxidase was quenched by incubating sections in 3% H_2_O_2_ in methanol for 30 mins at room temperature. Primary antibodies were detected using biotinylated goat anti-rabbit immunoglobulin secondary antibody (Vector Labs) in conjunction with an avidin-biotin peroxidase complex (Vector Labs) and 3’3, diaminobenzidine substrate (Vector Labs). All sections were counterstained in Gill’s haematoxylin, dehydrated, cleared and mounted in DPX (Sigma). Omission of primary antibody from the protocol and the use of concentration-matched isotype control antibodies were used as negative controls against non-specific binding of the detection system (see Supplementary Figure 4). Inclusion of a section of lung tissue, collected from patients undergoing surgical resection for lung cancer that had previously been deemed positive for a specific marker was used as a positive control (data not shown).

For sequential dual labelling of CD3 and PI3Kδ, slides of bronchial biopsy tissue were initially labelled with primary CD3 antibody overnight (diluted 1:2000; Dako, Ely, UK) and then alkaline phosphatase conjugated secondary antibody for 90 min (Vector Labs, Burlingame, USA), with staining detected using Vector Labs Blue AP Kit (Burlingame, USA). Slides were then blocked using 2.5% horse serum for 30 min, incubated overnight with PI3Kδ primary antibody (diluted 1:1500; Abcam), with staining detected using the ImmPRESS-HRP anti-rabbit IgG Polymer detection kit (Vector labs). Slides were counter stained with eosin.

**Image Analysis:**

Digital micrographs were obtained using a Nikon Eclipse 80i microscope (Nikon UK Ltd, Surrey, UK) equipped with a QImaging digital camera (Media Cybernetics, Marlow UK) and ImagePro Plus 6.0 software (Media Cybernetics). Quantification of individual cell counts and measurements of areas of interest were carried out using the ImagePro Plus 6.0 software.

Colour micrographs (RGBs) were separated into their red, green, blue (monochrome) constituents and analysis carried out on the blue channel. Using the count size feature in ImagePro Plus 6.0 software threshold values were selected to detect immunoreactivity above background. ImagePro Plus 6.0 software, used threshold values to quantify the number of cells positive and the area of positivity within a given area of interest (Epithelium or sub-epithelium). Quantification of pSTAT6 and PI3Kγ was determined as a percentage positive area of the specific area of interest due to the diffuse cytoplasmic staining pattern of these markers. Positively-stained subepithelial inflammatory cells were identified based on their colouration and morphology.  Sub-epithelial fibroblast cells with villous projections were excluded from the count.
