# Supplementary material for: Anti-inflammatory potential of PI3Kδ and JAK inhibitors in asthma patients
Source: Respir Res. 2016 Oct 4;17:124. doi: 10.1186/s12931-016-0436-2 (PMC5051065; doi:10.1186/s12931-016-0436-2)
Supplement: Additional file 3: Table S2. — In vitro enzymatic profiles for tofacitinib and PIK-294. (DOC 35 kb) [file 12931_2016_436_MOESM3_ESM.doc]

**Supplementary Table 2: *In vitro* enzymatic profiles for tofacitinib and PIK-294**

|  | **IC_50_ (nM)** | | | | **Reference** |
| --- | --- | --- | --- | --- | --- |
| **Tofacitinib** | **JAK1** | **JAK2** | **JAK3** | **Tyk2** |  |
|  | 3.2 | 4.1 | 1.6 | 34 |  |
| **PIK-294** | **p110α** | **p110β** | **p110δ** | **p110γ** |  |
|  | 10,000 | 490 | 10 | 160 |  |
