# Supplementary figures and images for: Anti-inflammatory potential of PI3Kδ and JAK inhibitors in asthma patients
Source: Respir Res. 2016 Oct 4;17:124. doi: 10.1186/s12931-016-0436-2 (PMC5051065; doi:10.1186/s12931-016-0436-2)

## Slide 1
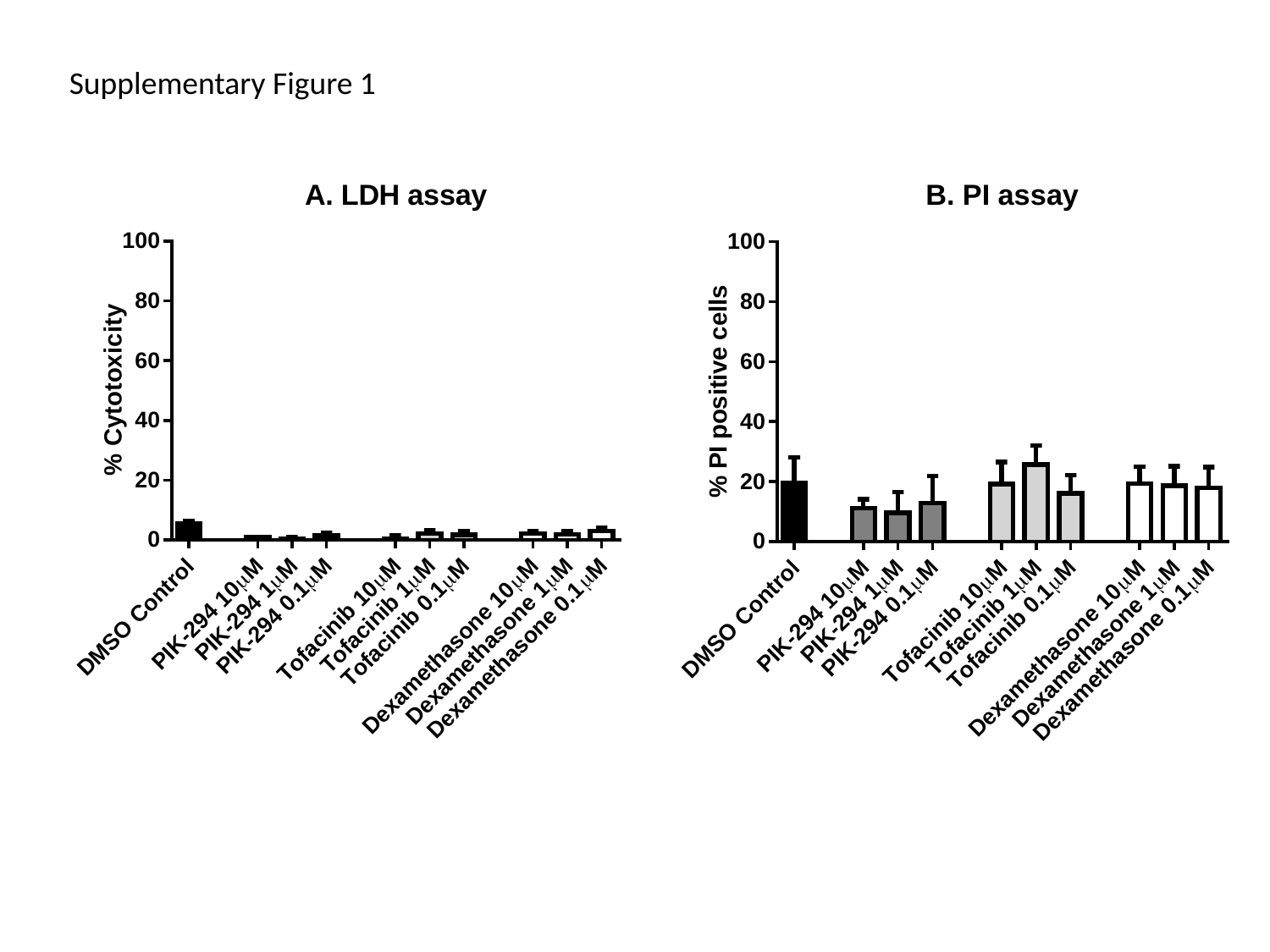

Supplementary Figure 1

Supplement: Additional file 4: Figure S1. — Cytotoxic effects of PIK-294, tofacinib and dexamethasone. PBMCs from healthy subjects (n = 3) were treated with PIK-294, tofacinib or dexamethasone for 1 h prior to TCR-stimulation for 72 h. Cytotoxicity was assessed by LDH assay (A) or propidium iodide flow cytometry assay (B). Cytotoxic effects were assessed by 1-way ANOVA with Dunnett’s post-hoc test against the no drug DMSO control. All non-significant. (PPTX 211 kb) [file 12931_2016_436_MOESM4_ESM.pptx]

## Slide 1
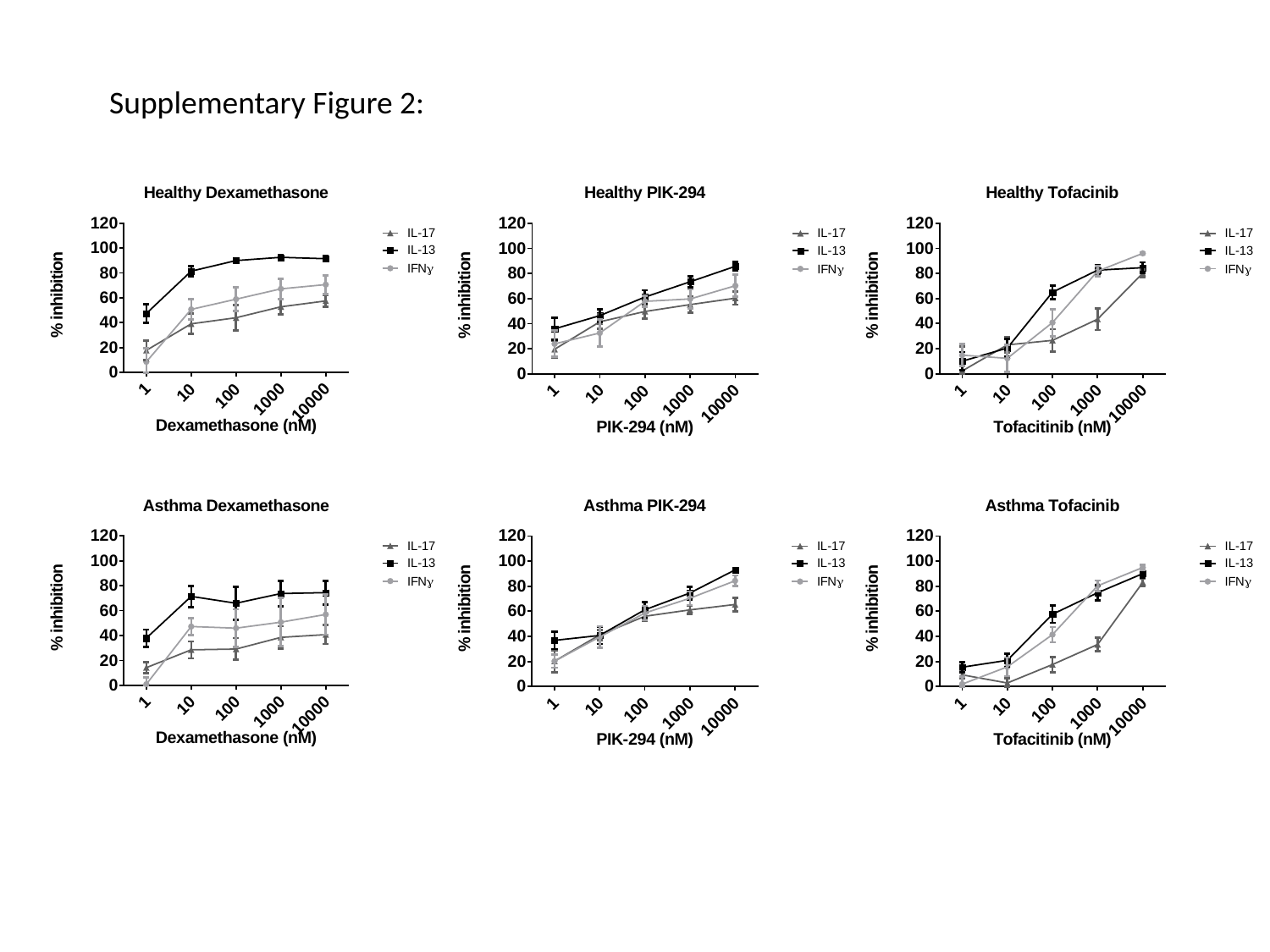

Supplementary Figure 2:

Supplement: Additional file 7: Figure S2. — Comparison of % inhibition of IFNγ, IL-13 and IL-17 for dexamethasone, tofacitinib and PIK-294. BAL cells from asthma patients (n = 12) and healthy subjects (n = 11) were treated with dexamethasone, tafacitinib or PIK-294 for 1 h before being stimulated with antibodies against CD3 and CD28 to induce a TCR-specific response. Cytokines were measured by ELISA and data is presented as the mean (+/− SEM) % inhibition in relation to the stimulated no drug control. (PPTX 742 kb) [file 12931_2016_436_MOESM7_ESM.pptx]

## Slide 1
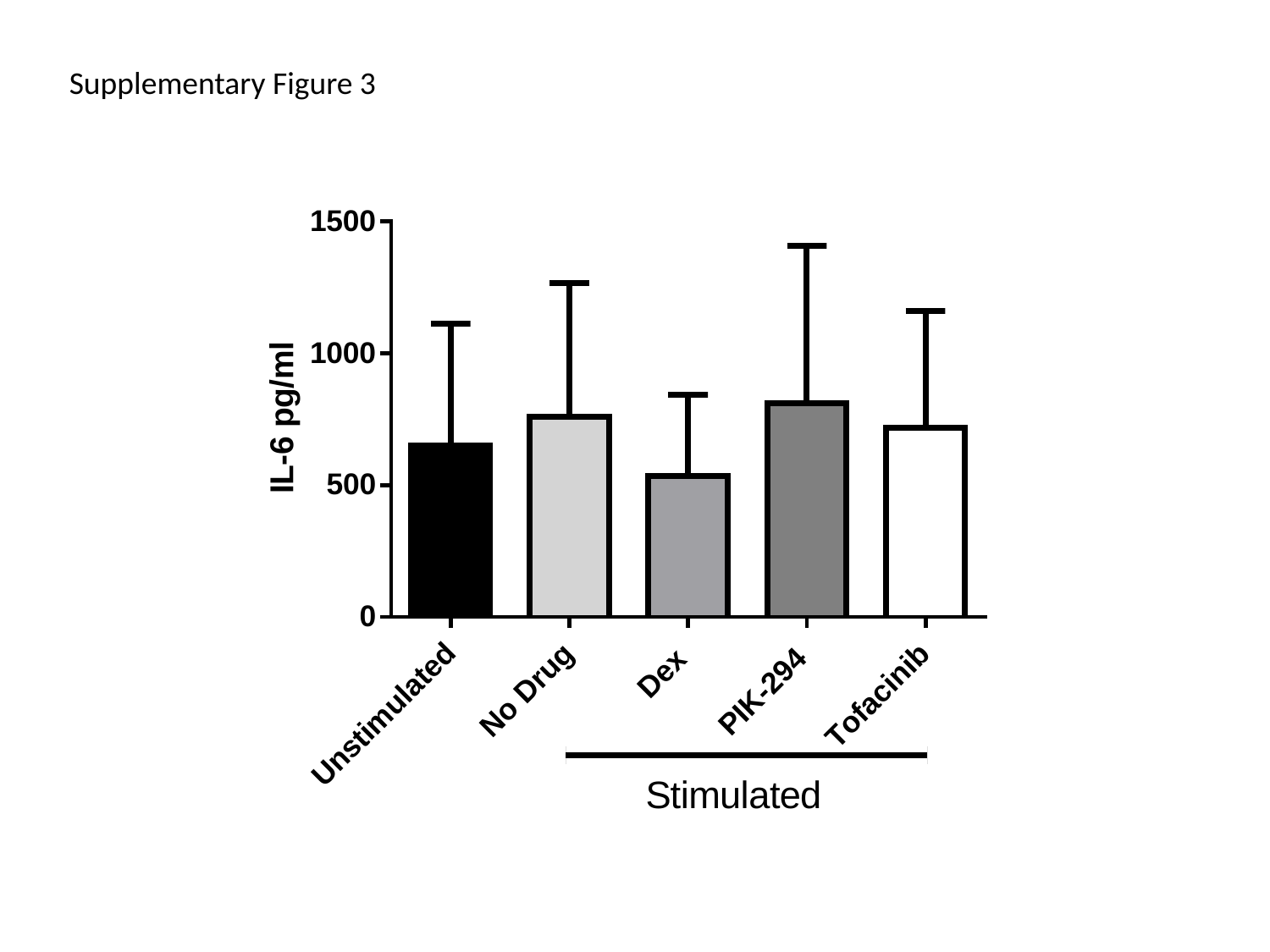

Supplementary Figure 3

Supplement: Additional file 9: Figure S3. — Effects of Dexamethasone, PIK-294 and tofacinib on IL-6 from TCR-stimulated BAL cells. BAL cells from asthma patients (n = 5) were treated with varying concentrations of dexamethasone with or without 100nM of tofacitinib for 1 h before being stimulated with antibodies against CD3 and CD28 to induce a TCR-specific response. IL-6 was measured by ELISA. Data is presented as mean +/− standard deviation. Drug effects were analysed by 1-way ANOVA: p = 0.22. (PPTX 89 kb) [file 12931_2016_436_MOESM9_ESM.pptx]
