# Supplementary material for: Anti-inflammatory potential of PI3Kδ and JAK inhibitors in asthma patients
Source: Respir Res. 2016 Oct 4;17:124. doi: 10.1186/s12931-016-0436-2 (PMC5051065; doi:10.1186/s12931-016-0436-2)
Supplement: Additional file 5: Table S3. — Immunohistochemistry retrieval buffers and antibody dilutions. (DOC 34 kb) [file 12931_2016_436_MOESM5_ESM.doc]

**Supplementary Table 3: Immunohistochemistry retrieval buffers and antibody dilutions.**

| **Primary antibody** | **Source (product code)** | **Stock concentration** | **Dilution** | **Final concentration** | **Retrieval buffer used** | **Secondary antibody** |
| --- | --- | --- | --- | --- | --- | --- |
| pSTAT1 | Cell Signalling (Cat No: 9167) | 59ug/ml | 1:200 | 295ng/ml | Trisodium citrate pH6.0 | Rabbit IgG |
| pSTAT3 | Cell Signalling (Cat No: 9145) | 111.5ug/ml | 1:800 | 139ng/ml | Tris-EDTA pH9.0 | Rabbit IgG |
| pSTAT5 | Cell Signalling (Cat No: 9359) | 163ug/ml | 1:200 | 815ng/ml | Tris-EDTA pH9.0 | Rabbit IgG |
| pSTAT6 | Abcam (Cat No: ab28829) | 1mg/ml | 1:400 | 2.5ug/ml | Tris-EDTA pH9.0 | Rabbit IgG |
| PI3kδ | Abcam ( Cat No:Ab1678) | 1mg/ml | 1:1600 | 0.625ug/ml | Trisodium citrate pH6.0 | Rabbit IgG |
| PI3kγ | Cell Signalling (Cat No: 4252) | 340ug/ml | 1:400 | 850ng/ml | Tris-EDTA pH9.0 | Rabbit IgG |
| pAkt | Cell Signalling (Cat no: 9271) | 10ug/ml | 1:50 | 200ng/ml | Tris-EDTA pH9.0 | Rabbit IgG |

v
