# Supplementary material for: Anti-inflammatory potential of PI3Kδ and JAK inhibitors in asthma patients
Source: Respir Res. 2016 Oct 4;17:124. doi: 10.1186/s12931-016-0436-2 (PMC5051065; doi:10.1186/s12931-016-0436-2)
Supplement: Additional file 6: Table S4. — Baseline and TCR-stimulated Cytokine levels in BAL cells. (DOC 35 kb) [file 12931_2016_436_MOESM6_ESM.doc]

**Supplementary Table 4: Baseline and TCR-stimulated Cytokine levels in BAL cells**.

| Cytokine | Condition | Cytokine levels (pg/ml) | | Comparison |
| --- | --- | --- | --- | --- |
|  |  | Healthy  (n=11) | Asthma (n=12) |  |
| IFNγ | Baseline | 16 (16-175) | 20.5 (16-1613) | P=0.39^a^ |
|  | Stimulated | 11,313 +/- 10,535 | 19,133 +/- 21,019 | P=0.28^b^ |
| IL-13 | Baseline | 4 (4-338) | 4 (4-10) | P=0.078^a^ |
|  | Stimulated | 130 +/- 100 | 370 +/- 292 | P=0.023^b^ |
| IL-17 | Baseline | 4 (4-54) | 4 (4-40) | P=0.88^a^ |
|  | Stimulated | 85 (17-3507) | 245 (32-900) | P=0.34^a^ |

Baseline levels of IFNγ, IL-13 and IL-17, and stimulated levels of IL-17 were non-parametrically distributed. Stimulated levels of IFNγ and IL-13 were normally distributed. Comparisons between healthy and asthma were by: ^a^Mann-Whitney test or ^b^unpaired T-test. Data is presented as either Median (range) or mean +/- standard deviation.
