# Supplementary material for: Anti-inflammatory potential of PI3Kδ and JAK inhibitors in asthma patients
Source: Respir Res. 2016 Oct 4;17:124. doi: 10.1186/s12931-016-0436-2 (PMC5051065; doi:10.1186/s12931-016-0436-2)
Supplement: Additional file 8: Table S5. — BAL Differential Cell Counts from historically collected samples. (DOC 29 kb) [file 12931_2016_436_MOESM8_ESM.doc]

**Supplementary Table 5: BAL Differential Cell Counts from historically collected samples.**

|  | **Neutrophil %** | **Macrophage %** | **Eosinophil %** | **Lymphocytes %** | **Epithelial Cells %** |
| --- | --- | --- | --- | --- | --- |
| **Asthma**  **(n=34)** | 1.0  (0.4 - 3.1) | 88.5  (83.4 - 93.9) | 0  (0 - 0.6) | 2.0  (1.2 - 3.3) | 3.5  (0.9 - 6.3) |
| **Healthy**  **(n=14)** | 1.1  (0.8 -2.4) | 94.8  (89.6 - 95.3) | 0  (0 - 2.4) | 1.6  (0.3 - 2.4) | 2.8  (1.5 - 3.75) |
|  | p=0.65 | p=0.08 | p=0.33 | p=0.22 | p=0.48 |

Data is presented as median (interquartile range). Comparisons between Asthma and Healthy were by Mann-Whitney test.
