# Supplementary material for: Anti-inflammatory potential of PI3Kδ and JAK inhibitors in asthma patients
Source: Respir Res. 2016 Oct 4;17:124. doi: 10.1186/s12931-016-0436-2 (PMC5051065; doi:10.1186/s12931-016-0436-2)
Supplement: Additional file 10: Figure S4. — Representative images for Immunohistochemistry negative controls. Bronchial biopsy tissue was stained using primary antibodies against PI3Kδ, PI3Kγ, pAKT, STAT1, STAT3, STAT5 and STAT6. Sequential sections were used for negative controls using either an isotype control negative antibody at the same concentration as the various primary antibodies, or with the omission of the primary antibody. Negative controls for each primary antibody were assessed in sequential tissue sections from n = 3 patients. Black bar represents 100 μm. (PPTX 487 kb) [file 12931_2016_436_MOESM10_ESM.pptx]

## Slide 1
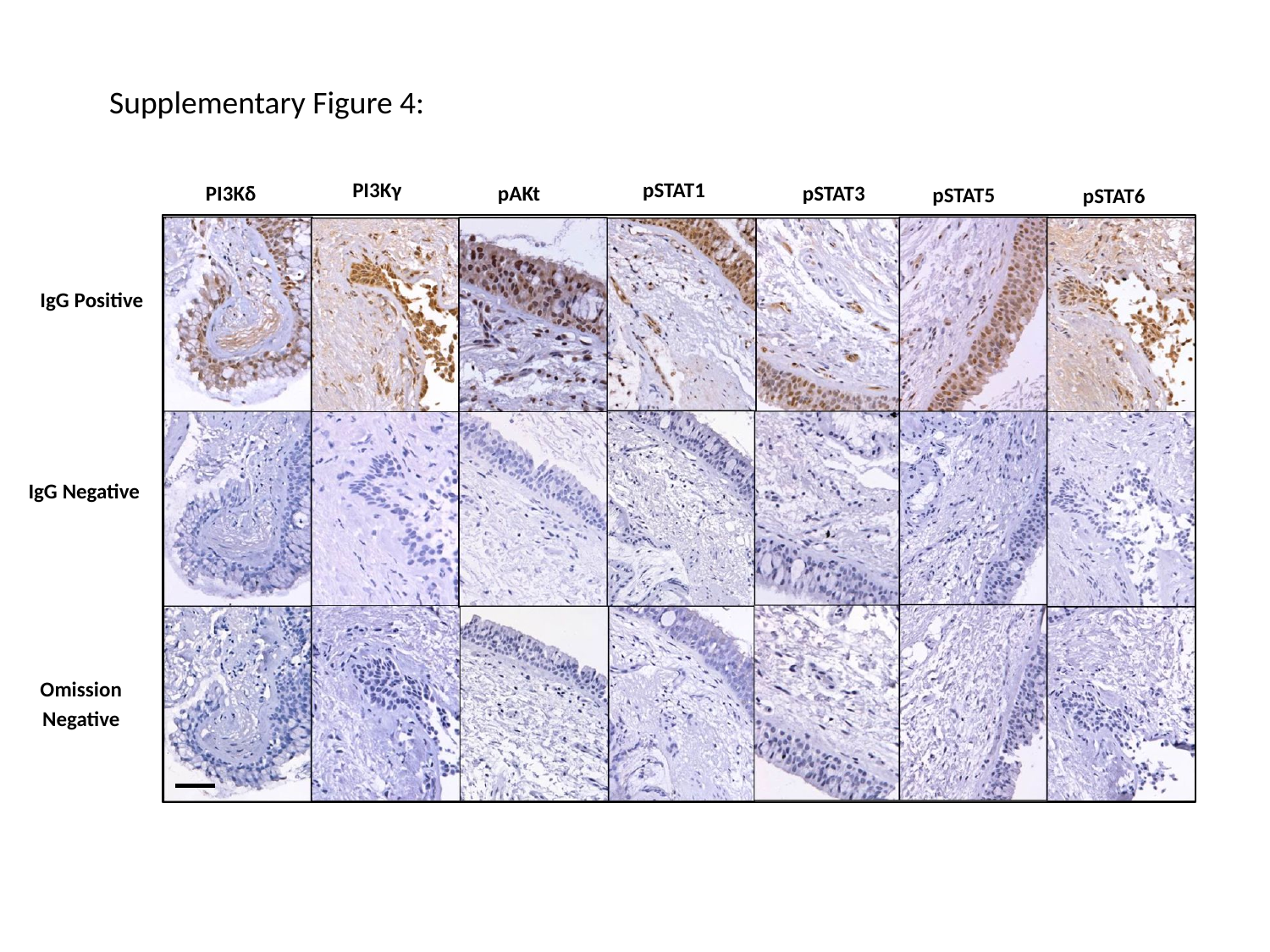

Supplementary Figure 4:
 PI3Kγ
 pSTAT1
 PI3Kδ
 pAKt
 pSTAT3
 pSTAT5
 pSTAT6
IgG Positive
IgG Negative
Omission Negative
