# Supplementary material for: Anti-inflammatory potential of PI3Kδ and JAK inhibitors in asthma patients
Source: Respir Res. 2016 Oct 4;17:124. doi: 10.1186/s12931-016-0436-2 (PMC5051065; doi:10.1186/s12931-016-0436-2)
Supplement: Additional file 11: Table S6. — Expression of pSTATs, pAKT, PI3Kγ and PI3Kγ by immunohistochemistry in bronchial biopsies. (DOC 41 kb) [file 12931_2016_436_MOESM11_ESM.doc]

**Supplementary Table 6: Expression of pSTATs, pAKT, PI3Kγ and PI3Kγ by immunohistochemistry in bronchial biopsies.**

| **Positive cells per mm^2^ of epithelium** | | |
| --- | --- | --- |
| **Target** | **Healthy** | **Asthma** |
| **pSTAT1** | 145 (0-958) | 383 (40-1246) |
| **pSTAT3** | 234 (0-1582) | 647 (0-2088) |
| **pSTAT5** | 10.9 (0-280) | 71.6 (17.2-1499) |
| **PI3Kδ** | 322 (11.1-808) | 747 (360-1515)*** |
| **pAKT** | 5891 +/- 2746 | 6244 +/- 1669 |
| **Positive cells per mm^2^ of subepithelium** | | |
| **Target** | **Healthy** | **Asthma** |
| **pSTAT1** | 22.1 (480-242) | 63.3 (19.0-526) |
| **pSTAT3** | 68.6 (5.6-759) | 67.0 (18.9-350) |
| **pSTAT5** | 10.0 (1.4-97.8) | 25.8 (2.1-440) |
| **PI3Kδ** | 119 (10.7-420) | 314 (145.5-1007)** |
| **pAKT** | 702 +/- 311 | 905 +/- 335 |
| **% positive epithelium** | | |
| **Target** | **Healthy** | **Asthma** |
| **pSTAT6** | 12.6 +/- 11.8 | 10.1 +/- 8.1 |
| **PI3Kγ** | 45.5 +/- 11.8 | 36.7 +/- 13.1 |
| **% positive subepithelium** | | |
| **Target** | **Healthy** | **Asthma** |
| **pSTAT6** | 0.80 +/- 0.81 | 0.44 +/- 0.33 |
| **PI3Kγ** | 6.1 +/- 3.4 | 6.0 +/- 5.0 |

pSTAT1, 3 & 5, and PI3Kδ expression was non-parametrically distributed and are presented as median (range), while pSTAT6 pAKT and PI3Kγ expression was normally distributed and are presented as mean ±SD. Expression in asthma tissue was compared with healthy by Unpaired T-tests or Mann-Whitney tests. **p<0.01; ***p<0.001
